# Alteration of Copper Fluxes in Brain Aging: A Longitudinal Study in Rodent Using ^64^CuCl_2_-PET/CT

**DOI:** 10.14336/AD.2017.1025

**Published:** 2018-02-01

**Authors:** Fangyu Peng, Fang Xie, Otto Muzik

**Affiliations:** ^1^Department of Radiology, and; ^2^Advanced Imaging Research Center, University of Texas Southwestern Medical Center, Dallas, TX75390, USA; ^3^Department of Pediatrics and; ^4^ Department of Radiology, Wayne State University, Detroit, MI 48202, USA

**Keywords:** Positron emission tomography, brain aging, Alzheimer’s disease, copper fluxes, glucose metabolism, copper-64 chloride

## Abstract

Brain aging is associated with changes of various metabolic pathways. Copper is required for brain development and function, but little is known about changes in copper metabolism during brain aging. The objective of this study was to investigate alteration of copper fluxes in the aging mouse brain with positron emission tomography/computed tomography using ^64^CuCl_2_ as a radiotracer (^64^CuCl_2_-PET/CT). A longitudinal study was conducted in C57BL/6 mice (n = 5) to measure age-dependent brain and whole-body changes of ^64^Cu radioactivity using PET/CT after oral administration of ^64^CuCl_2_ as a radiotracer. Cerebral ^64^Cu uptake at 13 months of age (0.17 ± 0.05 %ID/g) was higher than the cerebral ^64^Cu uptake at 5 months of age (0.11 ± 0.06 %ID/g, p < 0.001), followed by decrease to (0.14 ± 0.04 %ID/g, p = 0.02) at 26 months of age. In contrast, cerebral ^18^F-FDG uptake was highest at 5 months of age (7.8 ± 1.2 %ID/g) and decreased to similar values at 12 (5.2 ± 1.1 %ID/g, p < 0.001) and 22 (5.6 ± 1.1 %ID/g, p < 0.001) months of age. The findings demonstrated alteration of copper fluxes associated with brain aging and the time course of brain changes in copper fluxes differed from changes in brain glucose metabolism across time, suggesting independent underlying physiological processes. Hence, age-dependent changes of cerebral copper fluxes might represent a novel metabolic biomarker for assessment of human brain aging process with PET/CT using ^64^CuCl_2_ as a radiotracer.

Brain is a dynamic organ with changes of various metabolic pathways in adaptation to aging process [1]. Cerebral glucose metabolism is a reliable index of neural activity and may be used as a biomarker for functional imaging of brain function. Brain aging is characterized by decline of glucose metabolism, predominantly in the prefrontal cortex [2, 3]. 2-deoxy-2-[F-18]-fluoro-D-glucose (^18^F-FDG) positron emission tomograph (PET) is a useful tool for assessment of brain glucose metabolism, but use of ^18^F-FDG PET for differentiation of healthy brain aging from pathological brain aging is sometimes limited by normal variation of brain glucose metabolism and other confounding factors such as drug effects [4]. There are continued efforts to explore changes of various brain metabolic pathways, such as biometal metabolism or fluxes [5], for development of new biomarker for assessment of brain aging with PET imaging.

Copper is a nutritional metal required for brain development and function [6-10]. On the other hand, accumulation of excess copper in brain tissues could be harmful. Copper deficiency due to malfunction of ATP7A copper transporter encoded by mutated *ATP7A* gene [11-13] causes neurological disorder in Menkes disease [14], while accumulation of excess copper due to malfunction of ATP7B copper transporter encoded by mutated *ATP7B* gene [15-17] causes neurological disorder in Wilson’s disease (WD), or hepatolenticular neurodegeneration [18-21]. Significant progress has been made in understanding of cellular copper transport regulation by a delicate network of copper transporters and chaperons. However, systemic regulation of copper fluxes in brain aging and age-related neurodegenerative disorders remains poorly understood due to lack of a tool for longitudinal, real-time tracking of changes of brain copper fluxes with age.

Positron emission tomography (PET) is a useful tool for real-time tracking brain copper fluxes *in vivo* based on its high sensitivity and quantitative analysis capability [22]. Copper metabolism imbalance in *Atp7b* ^-/-^ knockout mouse model of Wilson’s disease was assessed with PET/CT using radioactive copper-64 chloride (^64^CuCl_2_) as a tracer [23, 24]. Recently, age-dependent changes of ^64^Cu radioactivity were detected in the brains of *Atp7b*^-/-^ knockout mouse model of WD with PET/CT [25]. Using this new ^64^CuCl2-PET/CT technique as a tool, this study aimed to conduct a longitudinal PET study to assess age-dependent changes of copper fluxes in the brains of C57BL/6 mice. Moreover, a concurrent longitudinal PET study using ^18^F-FDG) as a radiotracer [26] was performed to compare changes of copper fluxes with changes of glucose metabolism in mouse brain with aging. The findings of this pilot PET study for the first time demonstrated age-dependent changes of ^64^Cu radioactivity in the brains of mice orally administered with ^64^CuCl_2_ as a radiotracer, supporting potential use of cerebral ^64^Cu uptake as a biomarker for noninvasive assessment of brain aging and age-related neurodegenerative disorders using ^64^CuCl_2_-PET/CT as a tool.

## MATERIALS AND METHODS

### Small animals and Radiopharmaceuticals

C57BL/6 mice were purchased from Taconic Biosciences (Hudson, NY) and housed in the animal housing facility, UT Southwestern Medical Center at Dallas, with free access to copper-adequate food (AIN-93M Purified Rodent Diet, Dyets Inc., Bethlehem, PA) and drinking water. The tracer ^64^CuCl_2_ was purchased from Washington University (St Louis, MO), which was produced via ^64^Ni(p,n)^64^Cu using a biomedical cyclotron and supplied in the form of ^64^CuCl_2_ in 0.1M HCl solution. The specific activity of ^64^Cu was 6.9 ± 2.5 Ci/µmol. ^18^F-FDG was purchased from PetNet Solutions (Dallas TX). All small animal experiments were conducted according to a protocol approved by the Institutional Animal Care and Use Committee, UT Southwestern Medical Center, Dallas, TX.

**Figure 1.**
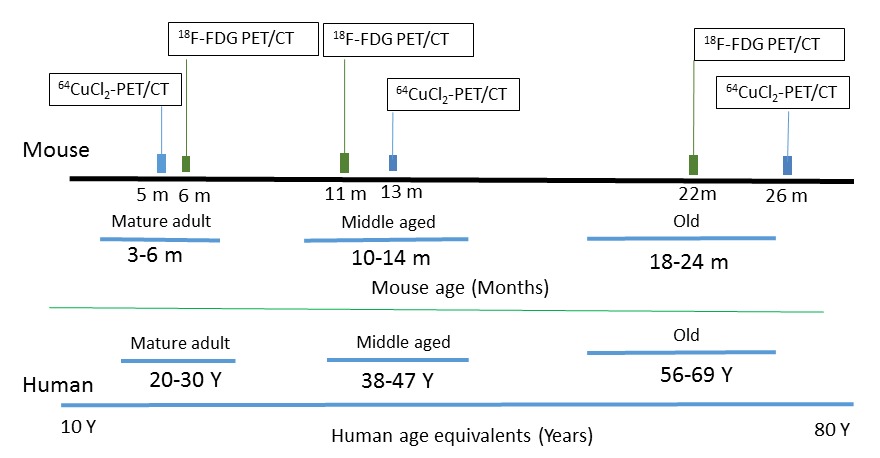
Schematic presentation of longitudinal study of glucose metabolism and copper fluxes in C57BL/6 mice with PET/CT Human age equivalents to mouse data were provided at the bottom.

**Table 1:** Whole body biodistribution of ^64^Cu (mean ± SD %ID/g) in C57BL/6 mice orally administered with ^64^CuCl_2_ by a longitudinal PET/CT.

	5 months (22 weeks)		13 months (59 weeks)		26 months (104 weeks)	
	2 h	24 h	2 h	24 h	2 h	24 h
Liver	1.15 ± 0.38	1.73 ± 0.38	1.25 ± 0.35	1.35 ± 0.07	2.13 ± 1.10	1.53 ± 0.41
heart	0.18 ± 0.08**	0.43 ± 0.07*	0.24 ± 0.01	0.29 ± 0.01*✝	0.50 ± 0.26**	0.56 ± 0.12✝
Muscle	0.06 ± 0.02	0.05 ± 0.05	0.06 ± 0.01	0.02 ± 0.02	0.09 ± 0.03	0.12 ± 0.12
Brain	0.11 ± 0.02	0.11 ± 0.06	0.14 ± 0.01	0.17 ± 0.05	0.12 ± 0.03	0.14 ± 0.06
Kidneys	0.53 ± 0.18	0.95 ± 0.13*	0.90 ± 0.42	0.81 ± 0.07✝	0.70 ± 0.53	0.48 ± 0.15*✝
Lungs	0.22 ± 0.08	0.44 ± 0.05	0.28 ± 0.03	0.36 ± 0.01	0.42 ± 0.31	0.46 ± 0.04

mean ± SD %ID/g, percentage of injected dose per gram of tissue

* p < 0.05;

✝ p < 0.05 for heart and kidney tracer concentration at 24h PO

** p < 0.05 for heart at 2h PO.

### Study design

A longitudinal study was performed to assess age-dependent changes of copper fluxes and glucose metabolism during normal aging process in a group of C57BL/6 mice (female, n = 5) by sequential ^64^CuCl_2_ and ^18^F-FDG PET/CT scans at 3 different time points (young, middle-aged, old) related to human age equivalents [Fig. 1].

**Table 2:** ^64^Cu uptake (mean ± SD %ID/g) in brains of C57BL/6 mice orally administered with ^64^CuCl_2_ measured by PET at 24h PO.

	5 months	13 months	26 months
Olfactory bulb	0.17 ± 0.06	0.26 ± 0.09	0.18 ± 0.04
Frontal cortex	0.17 ± 0.14	0.13 ± 0.02	0.17 ± 0.09
Posterior cortex	0.11 ± 0.05	0.12 ± 0.02	0.12 ± 0.05
Hippocampus	0.18 ± 0.05	0.19 ± 0.01	0.14 ± 0.02
Basal ganglia	0.16 ± 0.06	0.20 ± 0.02*	0.09 ± 0.03*
Thal/Hypothal	0.23 ± 0.10✝	0.21 ± 0.06*	0.11 ± 0.03*✝
Mid-brain	0.12 ± 0.03	0.11 ± 0.01	0.12 ± 0.02
Brain stem	0.12 ± 0.01✝**	0.20 ± 0.04**	0.19 ± 0.01✝
Cerebellum	0.12 ± 0.04	0.12 ± 0.01	0.15 ± 0.12

Thal, Thalamus; Hypothal, Hypothalamus; mean ± SD %ID/g, percentage of injected dose per gram.

* p < 0.05 for organs between 13 and 26 months.

✝ p < 0.05 for organs between 5 and 26 months.

** p < 0.05 for organs between 5 and 13 months

### PET/CT imaging

^64^CuCl_2_-PET/CT imaging of C57BL/6 mice was performed using a method described previously [23-25]. Briefly, the mice were anesthetized by 3% isoflurane in 100% oxygen (3 L/min) at room temperature, using an isoflurane vaporizer (Summit Anesthesia Solutions, Salt Lake City, UT). The mice were positioned in a spread-supine position on the imaging bed and subjected to inhalation of 2% isoflurane in 100% oxygen (3 L/min) during the PET/CT procedure. Static whole-body PET/CT imaging was then obtained for 15 minutes at 2 hour (h) post oral administration (PO) of ^64^CuCl_2_ (2 μCi (74 kBq)/g body weight) diluted in a volume of 25 µL normal saline (0.9% sodium chloride) using a blunted oral feeding tube, followed by static whole-body scan for 15 minutes at 24 h PO. PET/CT images were reconstructed using the ordered subsets expectation maximization 3D algorithm (OSEM3D), and data was analyzed using the Inveon Research Workplace (IRW) software (Siemens) which allows fusion of CT and PET image volumes. For ^18^F-FDG PET/CT imaging, C57BL/6 mice were subject to fasting for 10 to 12 hours prior to oral feeding of ^18^F-FDG (2 μCi (74 kBq)/g body weight) with a blunted feeding tube using a method modified from those described previously [25]. Acquisition and reconstruction of ^18^F-FDG PET/CT images were performed in analogy to the protocol used for ^64^CuCl_2_-PET/CT imaging described above.

**Figure 2.**
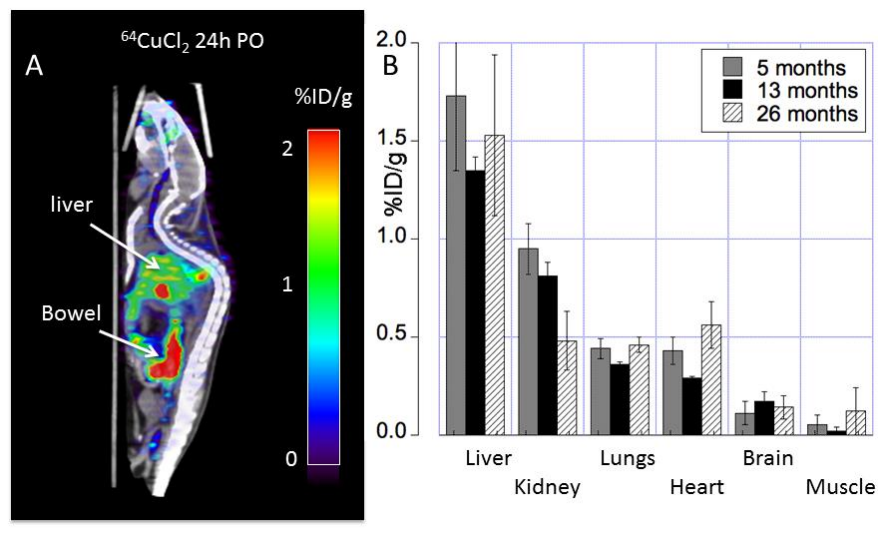
Biodistribution of ^64^Cu radioactivity in C57BL/6 mice orally administered with ^64^CuCl_2_ by PET/CT imaging (**A**) At 24h PO, prominent ^64^Cu radioactivity in the liver and gastrointestinal tracts were visualized on PET/CT images. Residual ^64^Cu radioactivity in oral cavity was also noted. (**B**) PET quantitative analysis determined ^64^Cu tissue distribution across different ages (5, 13 and 26 months of age) with ^64^Cu uptake in the heart and muscle at old age higher than ^64^Cu uptake at middle age. In contrast, ^64^Cu radioactivity in the kidneys at old age was lower than renal ^64^Cu radioactivity of C57BL/6 mice at young and middle age. Mean ± SD %ID/g: percentage of injected dose per gram tissue; PO: post oral administration of the tracer.

### Quantification of ^64^Cu and ^18^F-FDG radioactivity in the brain of C57BL/6 mice

^64^Cu and ^18^F-FDG radioactivity in the brains of C57BL/6 mice were analyzed using the Inveon Research Workplace (IRW) software (Siemens). In order to measure^64^Cu and ^18^F-FDG radioactivity in the different regions of the brains of C57BL/6 mice, 9 regions of interest (ROIs) were defined manually on the PET/CT images with reference to an MR imaging-based atlas of mouse brain anatomy [27] as described recently [25]. Regions included were: ROI 1 for olfactory bulb (OB), ROI 2 for frontal cortex (FC), ROI 3 for posterior cortex (PC), ROI 4 for hippocampus (HC), ROI 5 for basal ganglia (BG), ROI 6 for thalamus (T), ROI 7 for middle brain (MB), ROI 8 for brain stem (BS), ROI 9 for cerebellum (CB). The quantity of ^64^Cu and ^18^F-FDG radiotracer activity was obtained and recorded as a percentage of injected dose per gram tissue (%ID/g). Additionally, the mean ± standard deviation (SD) of %ID/g obtained from various ROIs was calculated and recorded for further statistical analysis.

### Statistical analysis

Initially, data was tested for normality using the Shapiro-Wilks test. Once normality was determined, parametric tests were performed in order to determine whether ^64^Cu uptake (mean ± SD %ID/g) differs among the young, middle age, and old C57BL/6 mice. Specifically, we applied a (2 x 3 x 9) repeated-measures ANOVA, with the three within-subject’s factors representing the scan time (2 or 24h PO), the age groups (young, middle age, and old) and the regions (ROI 1 - ROI 9). Following a significant overall test, pair-wise two-sample t-tests were conducted to determine significant differences among these groups. To correct for multiple comparisons, the adjusted Least Significant Difference (LSD) test (Sidak test) was performed for the post-hoc tests. Moreover, in order to determine whether the brain time course of ^64^Cu uptake at 24h PO differs from the brain time course of glucose uptake, we applied a (2 x 3 x 9) repeated-measures ANOVA, where the three within-subjects’ factors were the tracer (^64^Cu, ^18^F-FDG), the three time-points and the nine regions. A P value of less than 0.05 was considered as statistically significant.

**Table 3:** Whole body biodistribution of ^18^F-FDG (mean ± SD %ID/g) in C57BL/6 mice orally administered with ^18^F-FDG radiotracer by a longitudinal PET/CT.

	6 months (26 weeks)	11 months (44 weeks)	22 months (93 weeks)
Liver	1.25 ± 0.19✝	1.4 ± 0.49	2.0 ± 0.34✝
heart	1.85 ± 0.78✝	3.3 ± 1.15	3.4 ± 0.53✝
Muscle	0.76 ± 0.53	0.65 ± 0.39	1.3 ± 0.36
Whole brain	7.92 ± 1.19*✝	5.33 ± 1.63*	5.63 ± 1.16✝
Kidneys	1.98 ± 0.62	1.33 ± 0.21**	2.26 ± 0.31**
Lungs	1.20 ± 0.35✝	1.48 ± 0.82	1.8 ± 0.36✝

Mean ± SD %ID/g, percentage of injected dose per gram tissue.

* p < 0.05 for organs between 6 and 11 months.

✝ p < 0.05 for organs between 6 and 22 months.

** p < 0.05 for organs between 11 and 22 months

## RESULTS

### Biodistribution of ^64^Cu in C57BL/6 mice orally administered with ^64^CuCl_2_ at different ages

After oral administration of ^64^CuCl_2_ as a radiotracer, highest uptake of ^64^Cu was detected in the liver (1.73 ± 0.21 %ID/g) at 24h PO, followed by the kidneys (0.95 ± 0.13 %ID/g), with muscle showing lowest copper uptake (0.05 ± 0.05 %ID/g) in C57BL/6 mice at 5 months of age (Table 1, Fig. 2). Overall, ^64^Cu uptake in organs was higher at 24h PO as compared to 2h PO (p = 0.01). There was decrease of ^64^Cu uptake in the heart at middle age (0.29 ± 0.01%ID/g) compared to ^64^Cu uptake in the heart at young age (0.43 ± 0.07 %ID/g), followed by increase to 0.56 ± 0.12 %ID/g at old age. There was also interval increase of ^64^Cu uptake in the muscle from middle age (0.02 ± 0.02 %ID/g) to old age (0.12 ± 0.12 %ID/g) of C57BL/6 mice. In contrast, ^64^Cu radioactivity in the kidneys at old age (0.48 ± 0.15 %ID/g) was lower than renal ^64^Cu radioactivity of C57BL/6 mice at young (0.95 ± 0.13 %ID/g) and middle age (0.81 ± 0.07 %ID/g).

**Figure 3.**
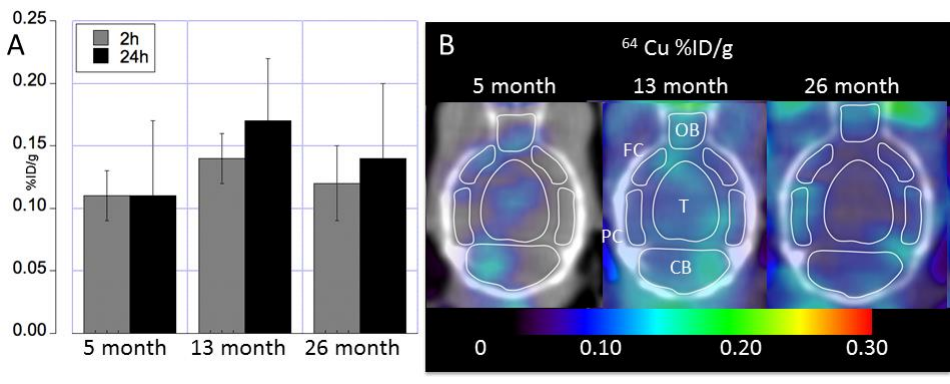
Whole brain regional ^64^Cu uptake at different ages assessed using longitudinal PET imaging (**A**) Whole brain ^64^Cu uptake (mean ± SD %ID/g) at 5, 13 and 26 months of age acquired at 2h and 24h post oral administration of the ^64^CuCl_2_ PET tracer. (**B**) Transaxial images showing regional ^64^Cu radioactivity (mean ± SD %ID/g) acquired 24h PO at 5, 13 and 26 months of age. Regions were shown for cerebellum (CB), Thalamus (T), olfactory bulb (OB), posterior cortex (PC), and frontal cortex (FC).

**Figure 4.**
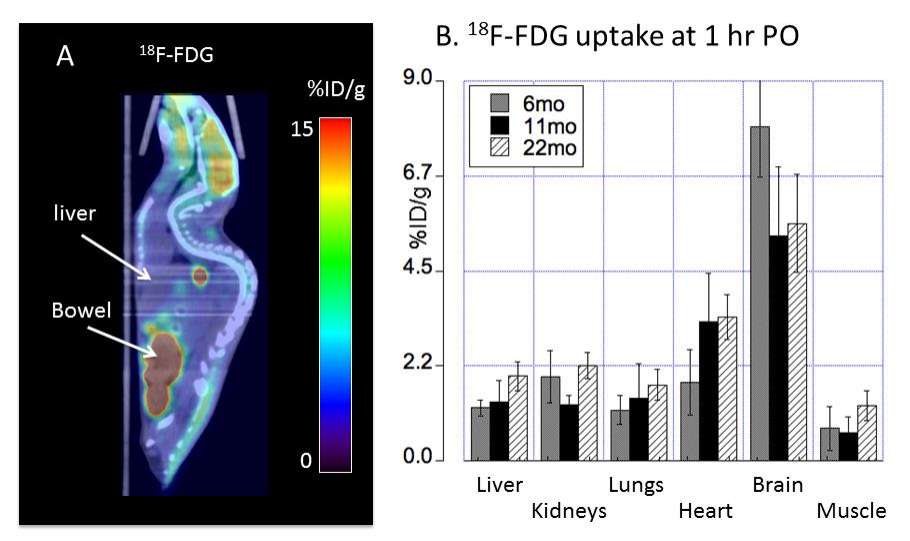
Biodistribution of ^18^F-FDG radioactivity (mean ± SD %ID/g) in C57BL/6 mice orally administered with ^18^F-FDG by PET/CT imaging (**A**) At 1h PO, prominent ^18^F-FDG radioactivity in the brain and gastrointestinal tracts was visualized on PET/CT images. Residual ^18^F-FDG was noted in oral cavity. (**B**) PET quantitative analysis demonstrated ^18^F-FDG tissue distribution across different ages (6, 11 and 22 months of age) with ^18^F-FDG uptake in the brain of middle and old age lower than ^18^F-FDG uptake in the brain of young adult C57BL/6 mice. Error bars represent SD; %ID/g: percentage of injected dose per gram tissue; PO: post oral administration of the tracer.

### Age-dependent changes of regional ^64^Cu radioactivity in the brains of C57BL/6 mice

We determined whole brain ^64^Cu uptake for images acquired at both 2h (p = 0.04) and 24h (p = 0.03) PO by longitudinal PET of C57BL/6 mice at 5, 13, 26 months of age (Table 1, Fig. 3A). Because images acquired at 24h PO displayed a greater dynamic range, all further analyses were performed using images acquired at 24h PO. Moreover, quantitative assessment of regional brain ^64^Cu tracer uptake showed significant differences between brain regions (Table 2, and Fig. 3B). Highest ^64^Cu uptake was determined in the olfactory bulb, followed by the brainstem and the basal ganglia, with cortical regions (frontal and parietal cortex) showing the lowest copper uptake (Table 2 and Fig. 3B). Taken together, our data indicates a dynamic time course of copper flux in the brain, increasing by more than 40% of ^64^Cu uptake from young to middle age, after which there is a decrease of ^64^Cu uptake to about 15% above values determined at young age.

### Biodistribution of ^18^F-FDG in C57BL/6 mice orally administered with ^18^F-FDG at different ages

Age-dependent changes of whole body biodistribution of ^18^F-FDG in C57BL/6 mice were determined by a longitudinal PET/CT imaging after oral administration of ^18^F-FDG radiotracer [Table 3, and Figure 4]. Expected variation of muscular ^18^F-FDG uptake was visualized in the C57BL/6 mice orally administered with ^18^F-FDG radiotracer. Physiologic whole brain ^18^F-FDG uptake at young age (7.92 ± 1.19 %ID/g) was significantly higher (p < 0.001) than uptake at both middle age (5.33 ± 1.63 %ID/g) and old age (5.63 ± 1.16 %ID/g), the latter two showing similar values (Table 3).

### Relationship between age-dependent changes of ^64^Cu and ^18^F-FDG radioactivity in the brains of C57BL/6 mice

**Figure 5.**
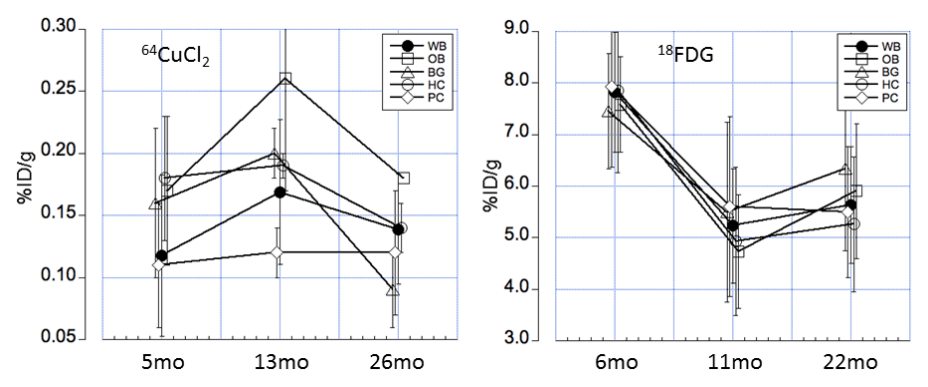
Correlative presentation of age-dependent changes of ^64^Cu uptake (left panel) and ^18^F-FDG uptake (right panel) determined using longitudinal PET analysis The figures demonstrate a significantly different time course of ^64^Cu and ^18^F-FDG uptake in the brain regions across the life span, indicating variable changes of copper fluxes and glucose metabolism in brain aging. Regions were shown for whole brain (WB), olfactory bulb (OB), basal ganglia (BG), posterior cortex (PC), and hippocampus (HC). Error bars represent SD; %ID/g, percentage of injected dose per gram tissue.

Age-dependent changes of regional brain ^18^F-FDG uptake was analyzed by PET quantitative analysis (Table 4) and compared with regional brain ^64^Cu uptake as described above (Table 2). In contrast to the observed low-level regional brain ^64^Cu uptake, high regional ^18^F-FDG uptake was determined in cortical, midbrain, and cerebellar regions (Table 4). Moreover, in addition to the large difference in overall tracer uptake, we determined differences in the temporal pattern between FDG and ^64^Cu brain uptake. Whereas ^64^Cu uptake increased from a low baseline value at young age to a maximum value at middle age and subsequently decrease to lower values at old age, ^18^F-FDG uptake in brain was highest at young age and decreased to lower values that were similar at both middle age and old age (Table 4, and Figure 5). This comparative analysis clearly demonstrates differences with respect to physiological uptake mechanisms of ^64^CuCl_2_ and ^18^F-FDG during the brain aging process.

**Table 4:** Regional ^18^F-FDG radioactivity (mean ± SD %ID/g) in the brains of C57BL/6 mice orally administered with ^18^F-FDG radiotracer measured with a longitudinal PET/CT.

	6 montsh (26 weeks)	11 months (44 weeks)	22 months (88 weeks)
Mid-brain	8.35 ± 1.23*✝	5.3 ± 1.72*	5.3 ± 1.04✝
Cerebellum	8.32 ± 1.33*✝	5.4 ± 1.39*	5.57 ± 1.01✝
Frontal cortex	7.58 ± 1.66*✝	5.23 ± 1.35*	5.17 ± 1.01✝
Posterior cortex	7.92 ± 1.56*✝	5.6 ± 1.74*	5.57 ± 1.27✝
Olfactory bulb	7.58 ± 0.92*✝	4.73 ± 1.10*	5.9 ± 1.31✝
Hippocampus	7.85 ± 1.59*✝	4.93 ± 1.43*	5.26 ± 1.31✝
Basal ganglia	7.45 ± 1.12	5.5 ± 1.74	6.33 ± 1.58
Thalamus/hypothalamus	7.05 ± 1.21*✝	5.1 ± 1.39*	5.27 ± 0.93✝
Brain stem	7.85 ± 0.92✝	5.8 ± 1.91	5.77 ± 1.33✝

* p < 0.05 between 6 and 11 months

✝ p < 0.05 between 6 and 22 months

## DISCUSSION

Brain aging process is associated with changes of various metabolic pathways. Development of radiotracers targeting brain metabolic changes is not only significant for a better understanding of physiology of healthy brain aging, but also significant for differentiation of healthy brain aging from pathological brain aging in AD and other neurodegenerative disorders in a content of brain aging. Copper is required for brain development and physiology although the role of copper in brain aging remains to be elucidated. Radionuclide ^64^Cu is a positron emitting copper isotope and ^64^CuCl_2_ was used as a radiotracer for noninvasive assessment of disturbance of cerebral copper fluxes in traumatic brain injury [28] and age-dependent changes of copper fluxes in *Atp7b*^-/-^ knockout mouse model of Wilson’s disease [25]. Applying ^64^CuCl_2_-PET/CT imaging in a mouse model of brain aging, we present data that show age-related differences in brain copper fluxes across the life span. Cerebral ^64^Cu radioactivity detected in middle-aged C57BL/6 mice was higher than the cerebral ^64^Cu radioactivity in young adult and middle-aged C57BL mice [Figure 2, Table 1 and 2]. More importantly, our findings demonstrate differences in both the time course as well as in the regional pattern between brain ^64^Cu and ^18^F-FDG uptake, indicating different time course of brain copper and glucose metabolism in brain aging. As copper functions as a nutritional metal required for development and function of brain, independent non-invasive assessment of copper fluxes in vivo might provide an important tool for the study of copper metabolic changes during both normal and pathological brain aging. Aging is a major risk factor for pathogenesis of AD [29]. Age-dependent changes of cerebral ^64^Cu radioactivity might be a useful biomarker for assessment of copper metabolic changes associated with AD with ^64^CuCl_2_ -PET/CT. Recently, Torres et al. used radioactive glyoxalbis(N4-methyl-3-thiosemicarbazonato)-64Cu(II) complex, 64Cu(II)-GTSM, as a radiopharmaceutical to examine intracranial copper transport in TASTPM transgenic AD mice [30]. Altered ^64^Cu trafficking was detected in the brains of TASTPM transgenic AD mice, with increased ^64^Cu concentration and faster brain ^64^Cu clearance compared to ^64^Cu trafficking in the brains of age-matched wild type control mice. Because ^64^Cu(II)-GTSM penetrates blood brain barrier and delivers ^64^Cu into brain tissue, ^64^Cu(II)-GTSM might be limited for evaluation of systemic regulation of gastrointestinal absorption and brain uptake of copper. It will be desirable to compare cerebral copper trafficking in TASTPM transgenic AD mice administered with ^64^Cu(II)-GTSM intravenously or ^64^CuCl_2_ orally for changes of gastrointestinal absorption of copper and subsequent copper influx to brain in AD.

Additional studies are necessary to address the following issues: (1) ROIs used for quantification of regional brain ^64^Cu radioactivity were subjected to errors in view of small size of mouse brain and limited spatial resolution of PET/CT; (2) Correlation of age-dependent changes of cerebral ^64^Cu radioactivity with changes of cognitive and behavioral activity of old C57BL/6 mice remains to be determined; (3) there may be difference of age-dependent changes of cerebral copper metabolism among different species; (4) Copper absorption may be affected by age and sex in humans [31]. However, our exploratory study only considered female C57BL/6 mice. Thus, further studies are required in male animals in order to determine whether age-dependent changes in copper flux differ between female and male C57BL/6 mice. Finally, the findings of this preclinical study of age-dependent changes of brain copper fluxes using C57BL/6 mouse model of brain aging need to be validated in human subjects.

The molecular mechanisms of age-dependent changes of brain copper fluxes observed in this exploratory study remain to be elucidated. Different amount of ^64^Cu uptake was detected in various regions of C57BL/6 mouse brain [Table 2], likely related to copper fluxes controlled by functional activity of copper transporters and chaperons [9, 32, 33]. It remains to be determined whether high ^64^Cu radioactivity in the thalamus/hypothalamus region of the C57BL/6 mice is correlated with high level of copper ions, because ^64^Cu radioactivity measured by PET represents dynamic flow of copper and may or may not represent regional content of brain tissue copper ions. The increased ^64^Cu uptake in the brains of middle-aged C57BL/6 mice compared with that in the brains of young adult C57BL/6 mice might reflect increased copper influx mediated by human copper transporter 1 (hCtr1) in response to physiological demand for higher copper ion concentration as a prerequisite for optimal functioning of copper-dependent enzymes. However, increased ^64^Cu radioactivity in middle-aged C57BL/6 mice could be the result of either increased retention or reduced efflux of copper mediated (by Atp7a or Atp7b) copper transporters or disturbances in intracellular copper transport mediated by copper chaperons [9, 32, 33]. Brain ^64^Cu uptake of old C57BL/6 mice was lower than that of middle-age C57BL/6 mice, possibly due to reduced demand for copper ions by copper-requiring enzymes in brain tissues or reduced copper influx mediated by hCtr1 or elevated copper efflux mediated by Atp7a or Atp7b. We believe that the creation of advanced imaging tools for longitudinal measurement of copper fluxes is highly relevant for the study of molecular mechanisms of age-dependent changes of brain copper fluxes regulated by copper transporters and chaperons, as well as the copper’s role in the pathogenesis of neurodegenerative disorders in the context of brain aging.

In summary, age-dependent changes of cerebral ^64^Cu radioactivity were detected in C57BL/6 mouse model of brain aging, showing increased ^64^Cu radioactivity in the brains of middle-aged C57BL/6 mice compared with cerebral ^64^Cu uptake in the young and old C57BL/6 mice. The findings support further investigation of age-dependent changes of copper fluxes in human brain aging and potential use of cerebral ^64^Cu uptake as a biomarker for differentiating healthy brain aging from pathological brain aging in AD and other neurodegenerative disorders using ^64^CuCl_2_-PET/CT as a tool.
